# On the Test of Silver for Arsenic

**Published:** 1818-04

**Authors:** John Prideaux


*For the London Medical and Physical Journal.*

### *On the Test of Silver for Arsenic;* by John Prideaux, Esq.

In your Number for January, is a letter from Mr. Hume, on the subject of his Silver Test for Arsenic; in which, after speaking rather disrespectfully of the objections to it, he puts the following questions:—

1. Is any test of arsenic infallible?

2. Is the production of the metal, in criminal cases, the only proof to be depended on?

3. Are phosphates, or phosphoric acid, always or ever present, in any of the animal fluids, in such a state as to yield a yellow precipitate with silver?

4. Is a decoction of onions capable of baffling the powers of chemistry to detect arsenic?

The reply to the second of these queries depends manifestly on that to the first. The fourth refers to a particular case, in which some detailed experiments, supposed to have proved the presence of arsenic, were repeated on infusion of onions with similar effects. The first and third, therefore, are the only ones that need discussion, and they will be best considered together.

The test for arsenic, being one on which life or death may frequently depend, is of infinitely greater importance than any other with which chemistry has to do.

The word "infallible," applied to a chemical test, has a two-fold signification: it must always indicate its re-agent, when present; and never produce deceitful appearances, by the action of other substances.

When suspected matter is found in the stomach, or its vomited contents, in the solid form, I believe Mr. Hume's test may be applied with the utmost confidence. But, when arsenic is dissolved in the liquid contents of the stomach, it may be doubted whether the same accurate results will ensue. I have tried the test in several instances where this poison had been given to dogs, and could never succeed in producing the yellow precipitate. Though, on mixing a solution of arsenic with the liquid taken from the human stomach after death, the precipitate appeared, when the quantity of mineral was *considerable*, and when the muriatic acid and some of the animal matters were got rid of, previous to the application of the test; yet, even in this way, success was not obtained with very minute proportions of the poison.

Has Mr. Hume ever successfully examined the liquid contents of a stomach, where arsenic was known to be the cause of death?—And can he point out a mode by which the same success may be generally depended on? He must see the necessity for this, when his test is to be considered a standard for the decision of juries, by which their "verdict* is to become unobjectionable." The ammoniaco-nitrate is not applicable, as it would entangle the arsenic in so overwhelming a quantity of muriate as to be hardly separable by any means.

On the other hand, in the liquid contents of a stomach, if a yellow precipitate be produced by the silver test, can we depend on its being the effect of arsenic? It may fairly be doubted, whether, since phosphate of soda does assuredly exist in the gastric fluid, its quantity may not be such, in some states of disease, as to affect the test in a slight degree; and a very little precipitate is all we can expect from arsenic in the state I am treating of. But phosphate of soda is used as a medicine, in quantity from one to two ounces at a dose, and is not very unlikely to be employed by a person, on the

---

* Mr. Hume's paper, Phil. Mag. September, 1812, p. 109.—See our Journal of the same date and page, Vol. xxviii., and also page 284 of the same Vol.; and Vol. xxix, p. 46.

attack of pain in the stomach, before medical aid be resorted to. Supposing the person to die of cholera morbus, it is by no means certain, that the medical attendants would be made acquainted with the salts having been employed; and the silver-test would be more probably affected by the contents of his stomach, than if he had swallowed a quantity of Fowler's solution, even greater than is requisite to produce death. It will, therefore, be necessary to have the means of distinguishing with certainty between these yellow precipitates, as obtained from the liquid contents of the stomach, when in such a state of disease as to have destroyed life. How are the methods pointed out in Mr. Hume's letter applicable?—Are they sufficient?—Would Mr. Hume himself venture "to swear to the presence of arsenic," by the application of his test to such a liquid?

Although I am satisfied, that, with solid arsenic, or suspected matter, nitrate of silver may be depended on in the hands of a chemist, yet I can by no means agree with Mr. Hume, that the verdict of juries, in cases of trial for poison, "will be rendered quite unobjectionable by its discovery," as a test. Besides the uncertainty, from which I cannot think it exempt, where no solid matter can be discovered, juries, and even judges, are very rarely qualified to appreciate the abilities of the analyst to whom the suspected matter may have been committed. A novice in chemistry might, without unjust design, occasion the death of an innocent person, by want of skill in investigation. It is true, if he were cross-examined by a chemist of experience, his testimony would lose its force; but such an examination cannot take place in a court of justice. The production of the metal is not liable to so much objection; for, although an unskilful hand would often fail when the mineral was present, yet he could not mistake any other metal for arsenic, and the risk of error would thus lie on the less injurious side. A quarter of a grain, and probably a much smaller quantity, will exhibit the metal very readily.

My objections to this test apply, however, only to its judicial employment. Whenever it is not dissolved in complicated animal or vegetable fluids, and where a mistake in its use leads to no dangerous consequences, its value cannot be questioned. Whether the ammoniaco-nitrate be an improvement, may admit of a doubt. In mineral analysis, on applying a liquid so strongly alkaline, other experiments may be necessary to distinguish the action of the alkali from that of the silver. May not some oxides, or subsalts, fall down, of such a colour as to indicate the presence of arsenic where it does not exist? When the alkali is applied first, we cannot be deceived.

I avoid entering into experimental details, as they may easily be exhibited by any chemist, and would uselessly occupy your pages. My object is, not to start futile objections, but to learn whether the known inconveniencies admit of being remedied. I have just read the papers to which Mr. Hume refers (except the first, which is well known,) with every attention, and find nothing in them applicable to the matter in question. Phosphate of soda is not once mentioned, nor does it appear that Mr. Hume was aware of its effect. It was discovered, if I remember right, since the publication of the last of those papers, by a gentleman of Oxford, and published in the Annals of Philosophy. I do not know that any mode of distinction was pointed out by Mr. Hume, or any one else, until after the trial at Launceston, in March last year; and, I must be forgiven for repetition, when I say, that I cannot think we shall be justified in influencing the opinion of a jury by tests of this kind, until the subject has been much more investigated.

*Plymouth; March* 6, 1818.

